# Genetic Polymorphism in a VEGF-Independent Angiogenesis Gene ANGPT1 and Overall Survival of Colorectal Cancer Patients after Surgical Resection

**DOI:** 10.1371/journal.pone.0034758

**Published:** 2012-04-04

**Authors:** Jingyao Dai, Shaogui Wan, Feng Zhou, Ronald E. Myers, Xu Guo, Bingshan Li, Xiaoying Fu, Juan P. Palazzo, Kefeng Dou, Hushan Yang, Jinliang Xing

**Affiliations:** 1 Department of Hepatobiliary Surgery, Xijing Hospital, The Fourth Military Medical University, Xi'an, China; 2 Division of Population Science, Department of Medical Oncology, Kimmel Cancer Center, Thomas Jefferson University, Philadelphia, Pennsylvania, United States of America; 3 Department of General Surgery, Tangdu Hospital, The Fourth Military Medical University, Xi'an, China; 4 State Key Laboratory of Cancer Biology, Cell Engineering Research Center & Department of Cell Biology, The Fourth Military Medical University, Xi'an, China; 5 Center for Human Genetics Research, Department of Molecular Physiology & Biophysics, Vanderbilt University, Nashville, Tennessee, United States of America; 6 Department of Pathology, Thomas Jefferson University, Philadelphia, Pennsylvania, United States of America; National Cancer Center, Japan

## Abstract

**Background:**

The VEGF-independent angiogenic signaling plays an important role in the development of colorectal cancer (CRC). However, its implication in the clinical outcome of CRC has not been reported. This study aimed to investigate the association between genetic variations in several major VEGF-independent signaling pathway genes and the overall survival of CRC patients.

**Methods:**

Seven single nucleotide polymorphisms (SNPs) in four important VEGF-independent angiogenic genes (*ANGPT1*, *AMOT*, *DLL4* and *ENG*) were genotyped in a Chinese population with 408 CRC patients.

**Results:**

One SNP, rs1954727 in *ANGPT1*, was significantly associated with CRC overall survival. Compared to patients with the homozygous wild-type genotype of rs1954727, those with heterozygous and homozygous variant genotypes exhibited a favorable overall survival with a hazard ratio (HR) of 0.89 (95% confidence interval [CI] 0.55–1.43, *P* = 0.623), and 0.32 (95% CI 0.15–0.71, *P* = 0.005), respectively (*P* trend = 0.008). In stratified analysis, this association remained significant in patients receiving chemotherapy (*P* trend = 0.012), but not in those without chemotherapy. We further evaluated the effects of chemotherapy on CRC survival that was stratified by rs1954727 genotypes. We found that chemotherapy resulted in a significantly better overall survival in the CRC patients (HR = 0.44, 95% CI 0.26–0.75, *P* = 0.002), which was especially prominent in those patients with the heterozygous genotype of rs1954727 (HR = 0.45, 95%CI 0.22–0.92, *P* = 0.028).

**Conclusion:**

Our data suggest that rs1954727 in *ANGPT1* gene might be a prognostic biomarker for the overall survival of CRC patients, especially in those receiving chemotherapy, a finding that warrants validation in larger independent populations.

## Introduction

Worldwide, colorectal cancer (CRC) is the third most common malignancy and the fourth leading cause of cancer death with an estimated 1,234,000 new cases and 608,000 deaths in 2008 [1]. CRC is a disease that is largely influenced by lifestyle and dietary factors [2], however, recent studies have suggested that inter-individual genetic variations may significantly affect the risk of CRC [3], [4], [5]. In addition, accumulating evidence, including those from our own studies, has also shown that single nucleotide polymorphisms (SNPs) may be used as surrogate biomarkers of the genetic background of CRC patients to predict therapeutic response and prognosis [6], [7], [8], [9].

Tumor angiogenesis, the generation of new blood vessels, is a crucial cellular process that influences tumor cell growth, invasion, local-regional recurrence, and metastatic spread of CRC, making it an attractive target for anticancer drug development [10].The tumor cell growth, invasion, and metastases are heavily influenced by the balance of functions of endogenous angiogenic and anti-angiogenic factors [10]. The vascular endothelial growth factors (VEGFs) and its receptors (VEGFRs) play a central role in the angiogenesis pathway [11]. The functional inhibitors of VEGF and VEGFRs, such as anti-VEGF neutralizing antibody and small molecules that block the tyrosine kinase activity of VEGFRs, have been approved as anti-angiogenesis therapies for many cancers, including CRC [12]. However, the wide resistance to the anti-angiogenic therapies targeting the VEGF pathway stimulated the search for treatments targeting the VEGF-independent angiogenesis pathway [12], [13], such as pro-angiogenic pathways mediated by angiopoietins/TIE-2 and Delta/Notch [14], [15], as well as anti-angiogenic pathways mediated by angiomotin and endoglin proteins [16], [17].

Genetic variations in the *VEGF* gene have been reported to modulate VEGF gene expression [18], [19], [20]. They have also been associated with the etiology and clinical outcomes of CRC [21], [22], [23]. However, although basic studies have revealed an essential role of VEGF-independent pathway genes in the etiology and clinical outcome of CRC, no study has been reported on the association between the genetic variations of these genes and CRC prognosis. The aim of the current pilot study was to evaluate the association between SNPs in several major VEGF-independent angiogenic pathway genes with the overall survival of CRC patients.

## Materials and Methods

### Ethics

This study was approved by the institutional review boards of the Fourth Military Medical University. A written informed consent with a signature was obtained for each patient.

### Patient population and clinical data collection

This study was focused on the VEGF-independent angiogenesis gene SNPs as potential predictors of CRC prognosis. Therefore, all subjects included in this study had histopathologically confirmed CRC. Originally, 496 CRC patients were recruited between February 2006 and April 2010 from the Departments of General Surgery in the Xijing Hospital and the Tangdu Hospital that are affiliated with the Fourth Military Medical University in Xi'an, China. There were no recruitment restrictions on age, gender, and cancer stage. All CRC patients had no previous history of other cancers, chemotherapy, or radiotherapy. All CRC patients were newly diagnosed and histopathologically confirmed as having adenocarcinoma. For the purposes of this study, 88 patients were excluded, for the following reasons: did not undergo surgery or only received palliative operation (26 patients), had incomplete clinical information or lacked follow-up (48 patients), died within one month of surgery (6 patients) and had poor quality and/or quantity of DNA sample (8 patients). Finally, 408 surgically resected patients with complete clinical and follow up data as well as high quality DNA samples were included in the present study. All patients were Han Chinese.

### Demographic and clinical data

Detailed demographic and clinical information was collected through in-person interview, medical chart review, or consultation with treating physicians. The demographic data collected include age, gender, ethnicity, smoking status, drinking status, and body mass index (BMI). The clinical data collected include time of diagnosis, time of surgery and/or chemotherapies, time of death, tumor stage, differentiation, location site, lymph node invasiveness, treatment protocol, and serum CEA (carcinoembryonic antigen). A standard follow-up was performed by a trained clinical specialist through on-site interview, direct calling, or medical chart review. The latest follow-up data in this analysis was obtained in February 2011. For patient enrolled after August 2008, 5-ml of blood was available for genomic DNA extraction using the E.Z.N.A. Blood DNA Midi Kit (Omega Bio-Tek, Norcross, GA). For patients enrolled before August 2008, genomic DNA was extracted from approximately 100 mg of adjacent normal tissues obtained by a pathologist after surgery using the E.Z.N.A. tissue DNA Kit (Omega Bio-Tek).

### SNP selection and genotyping

Four major genes in the VEGF-independent angiogenic pathway were studied, including *AGNPT1* which encodes a TIE2 agonist that stimulates the PI3K-Akt signaling pathway as a survival signal and stabilizes blood vessels, *DLL4* (Delta-like 4 ligand), which encodes a protein that is secreted from endothelial cells and promotes angiogenesis by suppressing non-functional sprouting of vascular endothelial cells, *AMOT* (Angiomotin) which encodes an angiostatin inhibitor that mediates tube formation and migration of endothelial cells toward growth factors during the formation of new blood vessels, and *ENG* (Endoglin) which encodes a component of the transforming growth factor beta receptor complex involved in angiogenesis, cardiovascular development, and vascular homeostasis. Potentially functional SNPs, in functional regions including promoters, exons, and untranslated regions (UTRs) were selected using a set of web-based SNP selection tools (freely available at http://snpinfo.niehs.nih.gov/snpfunc.htm), by which one can select SNPs based on linkage disequilibrium (LD) and predicted functional characteristics of both coding and non-coding SNPs. The 5′ and 3′ flanking regions were arbitrarily set at 1000 bp for all genes. Only validated SNPs were selected and SNPs with minor allele frequency (MAF) <5% in Asian population were excluded. In the case of multiple potentially functional SNPs within the same haplotype block (defined by the linkage coefficient r^2^>0.8), only one SNP was included. Finally, a total of seven SNPs were identified for the four genes, including two SNPs in *ANGPT1* (rs1954727 and rs9297395), three SNPs in *AMOT* (rs2286064, rs2286063, rs640009), one SNP in *DLL4* (rs12439845) and one SNP in *ENG* (rs7865146). Genotyping was performed using the Sequenom iPLEX genotyping system (Sequenom Inc, CA). Laboratory personnel conducting genotyping were blinded to patients' information. The average call rate for the genotyping was 99.4%. Strict quality control measures were implemented during genotyping with over 99% concordance between samples genotyped in duplicate.

### Statistical analysis

The endpoint evaluated in this study was overall survival, defined as the time from initial surgery to death or last follow-up. Hazard ratio (HR) and 95% confidence interval (95% CI) were estimated using a multivariate Cox proportional hazards model, adjusting for age, gender, smoking status, drinking status, BMI, tumor position, tumor differentiation, tumor stage, and chemotherapy, where appropriate. Haplotype/diplotype was determined using the HelixTree software (Golden Helix, Inc.), and HR was estimated by multivariate Cox proportional hazards model, using the haplotype/diplotype containing all wild-type alleles as reference. The tests for interactions between SNPs and DNA source were conducted by including a cross-product term into the Cox proportional hazards model. Log-rank test was used to assess the differences of overall survival between different patient groups. SAS statistical package (SAS software version 9.2, Cary, NC) was used for the analyses. All *P* values in this study were two-sided. *P*≤0.05 was considered the threshold of statistical significance.

## Results

### Patients Characteristics

A total of 408 surgically resected CRC patients were included in this study, with an average age of 59.4 years (range, 22 to 90 years) and mean BMI of 22.7 (range, 15.8 to 32.9). Among the patients, 56.4% (n = 230) were males and 46.6% (n = 178) were females. The majority of patients were never smokers (70.8%) and never drinkers (89.5%). The percentage of the patients with colon cancer (47.1%) was slightly lower than rectum cancer patients (52.9%). About 47.1% of patients had stage II tumor, whereas stage 0, I, III and IV tumors were present in 2.0%, 14.2%, 27.2% and 9.6% of patients, respectively. Approximately 66.4% of patients had moderately differentiated tumors and 78.2% patients were treated with chemotherapy after surgery. In a median follow-up time of 22.9 months, approximately 23.0% (n = 94) of the patients were deceased as of the last follow up (February 2011) (**Table 1**).

**Table 1:** Demographic and clinicopathological characteristics of patients with CRC.

Variables	Number of patients (%), N = 408
**Age, average(range) (in years)**	59.4 (22–90)
**Body mass index, mean (range)**	22.7(15.8–32.9)
**Gender**	
Male	230 (56.4)
Female	178(46.6)
**Smoking status**	
Ever	119 (29.2)
Never	289 (70.8)
**Drinking status**	
Ever	43 (10.5)
Never	365 (89.5)
**Tumor position**	
Colon	192 (47.1)
Rectum	216 (52.9)
**Tumor stage**	
0	8 (2.0)
I	58 (14.2)
II	192 (47.1)
III	111 (27.2)
IV	39 (9.6)
**Tumor differentiation**	
Poor	37 (9.1)
Moderate	271 (66.4)
Well	100 (24.5)
**Chemotherapy**	
Yes	319 (78.2)
No	89 (21.8)
**Death**	
Yes	94 (23.0)
No	314 (77.0)

### Association between individual SNPs and overall survival in CRC patients

**Figure 1:**
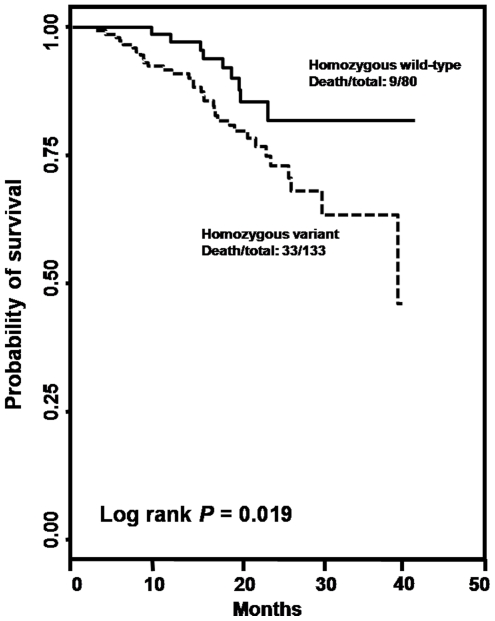
Kaplan-Meier curves and log rank tests for rs1954727.

The associations of the seven selected SNPs with CRC survival were analyzed using univariate and multivariate analyses adjusting for age, gender, smoking status, drinking status, BMI, tumor position, tumor differentiation, tumor stage, and chemotherapy. The results were summarized in **Table 2**. Among them, one SNP, rs1954727 located in the 3′ UTR region of the *ANGPT1* gene, was significantly associated with the overall survival in CRC patients in both univariate and multivariate analyses. In multivariate analysis, compared to the homozygous wild-type genotype (WW), the heterozygous (WV) and homozygous variant genotype (VV) of rs1954727 was associated with an HR of 0.89 (95% CI 0.55–1.43, *P* = 0.623) and 0.32 (95% CI 0.15–0.71, *P* = 0.005), respectively, with a significant dose-response effect observed (*P* trend = 0.008, **Table 2**). Log rank test indicated a significant difference in overall survival between the wild-type and homozygous variant genotypes of rs1954727 (Log rank *P* = 0.019, Figure 1). These results indicated that rs1954727 was an independent predictor of CRC survival in our study population. In addition, we randomly selected 50 patients included in this study with both blood and normal tissues available and genotyped all the seven SNPs in both blood and tissue DNAs for these patients. The genotyping results were 100% concordant between blood and tissue DNA samples. Furthermore, for each of the seven SNPs, we conducted a test for interaction (heterogeneity) between the SNP and DNA source and did not identify any significant interaction (*P* for interaction for overall survival ranges from 0.143 for rs1954727 to 0.651 for rs2286063, data not shown). Taken together, these lines of evidence indicated that the different DNA sources did not affect the findings identified in this study.

**Table 2:** Association of SNPs with overall survival in CRC patients.

Gene	Region	SNP	Genotype^a^	Death/total	HR (95% CI)^b^	*P* value	HR (95% CI)^c^	*P* value
*ANGPT1*	3UTR	rs1954727	WW	33/133	1(reference)		1(reference)	
			WV	52/192	1.25(0.80–1.93)	0.324	0.89(0.55–1.43)	0.623
			VV	9/80	**0.42(0.20–0.89)**	**0.023**	**0.32(0.15–0.71)**	**0.005**
			*P* for trend			0.084		**0.008**
*ANGPT1*	Flanking 5UTR	rs9297395	WW	79/360	1(reference)		1(reference)	
			WV	12/32	1.74(0.95–3.19)	0.075	1.20(0.56–2.54)	0.641
			VV	3/13	1.11(0.35–3.53)	0.855	0.83(0.24–2.92)	0.771
			*P* for trend			0.232		0.900
*AMOT*	3UTR	rs2286064	WW	81/367	1(reference)		1(reference)	
			WV	8/22	1.24(0.57–2.70)	0.587	2.02(0.85–4.81)	0.110
			VV	5/18	1.10(0.44–2.71)	0.840	0.58(0.21–1.59)	0.290
			*P* for trend			0.678		0.613
*AMOT*	Coding	rs2286063	WW	64/277	1(reference)		1(reference)	
			WV	14/52	1.29(0.72–2.31)	0.384	1.50(0.75–3.02)	0.253
			VV	16/78	0.98(0.57–1.70)	0.942	1.27(0.71–2.28)	0.418
			*P* for trend			0.879		0.375
*AMOT*	Flanking 5UTR	rs640009	WW	50/201	1(reference)		1(reference)	
			WV	19/85	0.95(0.56–1.62)	0.861	1.21(0.58–2.56)	0.609
			VV	25/120	0.87(0.54–1.41)	0.567	0.85(0.49–1.47)	0.550
			*P* for trend			0.570		0.951
*DLL4*	Flanking 5UTR	rs12439845	WW	42/189	1(reference)		1(reference)	
			WV	40/163	0.99(0.64–1.54)	0.982	0.93(0.58–1.49)	0.760
			VV	12/51	0.93(0.49–1.76)	0.820	0.60(0.28–1.29)	0.190
			*P* for trend			0.850		0.118
*ENG*	Flanking 5UTR	rs7865146	WW	19/74	1(reference)		1(reference)	
			WV	44/200	0.98(0.56–1.69)	0.936	0.63(0.34–1.17)	0.146
			VV	31/130	1.07(0.60–1.92)	0.819	1.09(0.55–2.19)	0.800
			*P* for trend			0.778		0.818

Note: The significant *P* values (≤0.05) were in bold.

a WW, homozygous wild-type genotype; WV heterozygous genotype; VV, homozygous variant genotype.

b Univariate analysis.

c Adjusted for age, gender, smoking status, drinking status, BMI, tumor position, tumor differentiation, tumor stage and chemotherapy.

### Association of rs1954727 with overall survival of CRC patients stratified by host characteristics

We further analyzed the effect of rs1954727 on overall survival in CRC patients stratified by demographic and clinical characteristics. As shown in **Table S1**, the significant better overall survival conferred by rs1954727 was observed in older patients (HR = 0.12, 95% CI, 0.03–0.60, *P* = 0.010), male patients (HR = 0.20, 95% CI, 0.05–0.75, *P* = 0.017), never smokers (HR = 0.28, 95% CI, 0.11–0.73, *P* = 0.009), never drinkers (HR = 0.35, 95% CI, 0.15–0.84, *P* = 0.018), and the patients with lower education level (HR = 0.28, 95% CI, 0.08–0.94, *P* = 0.040), with chemotherapy (HR = 0.33, 95% CI, 0.14–0.79, *P* = 0.012), with colon cancer (HR = 0.09, 95% CI, 0.02–0.40, *P* = 0.002), with poor and moderate tumor differentiation (HR = 0.32, 95% CI, 0.13–0.78, *P* = 0.012), with stage 3 or 4 tumors (HR = 0.16, 95% CI, 0.04–0.65, *P* = 0.011), and with both lower or higher BMI (HR = 0.20, 95% CI, 0.04–0.97, *P* = 0.045, or HR = 0.32, 95% CI, 0.11–0.92, *P* = 0.035, respectively). The significant dose-dependent effect conferred by rs1954727 was evident in patients who were older (*P*_trend_ = 0.006), male (*P*_trend_ = 0.012), never smoker (*P*_trend_ = 0.021), never drinker (*P*_trend_ = 0.025), and patients with higher BMI (*P*_trend_ = 0.025), with chemotherapy (*P*_trend_ = 0.012), with tumor position of colon cancer (*P*_trend_ = 0.012), with poor and moderate tumor differentiation (*P*_trend_ = 0.016), and with advanced tumor stage of 3–4 (*P*_trend_ = 0.003). Among the 319 patients receiving chemotherapy, 308 (96.6%) received the FOLFOX regimen, including folinic acid (FOL), fluorouracil (F) and Oxaliplatin (OX). Restricting the analysis to those patients receiving the FOLFOX yielded similar results to those of the current analysis (data not shown).

### Modulating effects of chemotherapy on CRC overall survival by rs1954727

Because the majority of CRC patients in this study were treated with chemotherapy, we further assessed the modulating effects on the association between chemotherapy and overall patient survival, stratified by rs1954727. As shown in Table 3, patients receiving chemotherapy had a significantly better overall survival with an HR of 0.44 (95% CI, 0.26–0.75, *P* = 0.002), compared to patients without chemotherapy. This significant effect on patient survival conferred by chemotherapy was only observed in patients with the heterozygous genotype of rs1954727 in the stratified analysis (HR = 0.45, 95% CI 0.22–0.92, *P* = 0.028), but not in those carrying the homozygous wild-type or variant genotype.

**Table 3:** Modulating effects of chemotherapy on colorectal cancer overall survival by rs1954727 genotypes.

SNP and variables^a^	Death/total	HR (95% CI)^b^	*P* value
**In all patients**			
No chemotherapy	22/89	1(reference)	
Chemotherapy	72/319	**0.44(0.26–0.75)**	**0.002**
**In patients with WW genotype of rs1954727**			
No chemotherapy	7/31	1(reference)	
Chemotherapy	26/102	0.45(0.16–1.27)	0.132
**In patients with WV genotype of rs1954727**			
No chemotherapy	14/43	1(reference)	
Chemotherapy	38/149	**0.45(0.22–0.92)**	**0.028**
**In patients with VV genotype of rs1954727**			
No chemotherapy	1/15	1(reference)	
Chemotherapy	8/65	0.19(0.01–4.25)	0.297

Note: The significant *P* values (≤0.05) were in bold.

a WW, homozygous wild-type genotype; WV heterozygous genotype; VV, homozygous variant genotype.

b Adjusted for age, gender, smoking status, drinking status, BMI, tumor position, tumor differentiation, tumor stage, and chemotherapy, where appropriate.

### Haplotype and diplotype of *ANGPT1* gene and CRC overall survival

The haplotype and diplotype analyses were conducted to evaluate the combined effect of the two SNPs in the *ANGPT1* gene on CRC overall survival. As shown in Table 4, there were four haplotypes in the order of rs9297395 and rs1954727 (W_W: 52.5%, W_V: 42.2%, V_W: 4.3%, and V_V: 1.0%) and three diplotypes (W_W-W_W: 28.8%, W_W-W_V: 44.3%, and W_V-W_V: 19.7%) with a frequency >1% in the study population. When compared with the haplotype containing the wild-type allele for the two SNPs (W_W), patients with the haplotype of W_V exhibited a significantly better overall survival (HR = 0.59, 95%CI 0.42–0.83, *P* = 0.002), which was consistent with the main effect analysis of the rs1954727 SNP. For diplotype analysis, when compared with the diplotype containing the homozygous wild-type genotype for both SNPs (W_W-W_W), diplotype containing one copy of the W_V haplotype (W_W-W_V) and two copies of W_V haplotype (W_V-W_V) exhibited a progressively improved overall survival with an HR of 0.74 (95% CI 0.44–1.24, P = 0.247) and 0.30 (95% CI 0.13–0.67, *P* = 0.004), respectively. We also performed the haplotype and diplotype analyses for the three SNPs in *AMOT* gene, however, no significant findings were observed (data not shown).

**Table 4:** Halpotype and diplotype analyses of *ANGPT1* SNPs and CRC survival.

Group^a^	Frequency	Death/total	HR (95%CI)^b^	*P* value
SNP: rs9297395-rs1954727				
Haplotype				
W_W	52.5%	102/405	1(reference)	
W_V	42.2%	60/326	**0.59(0.42–0.83)**	**0.002**
V_W	4.3%	25/33	0.75(0.34–1.62)	0.459
V_V	1.0%	2/8	1.63(0.38–6.96)	0.512
Diplotype				
W_W-W_W	28.8%	28/111	1(reference)	
W_W-W_V	44.3%	42/171	0.74(0.44–1.24)	0.247
W_V-W_V	19.7%	9/76	**0.30(0.13–0.67)**	**0.004**

Note: The significant *P* values (≤0.05) were in bold.

a WW, homozygous wild-type genotype; WV heterozygous genotype; VV, homozygous variant genotype.

b Adjusted for age, gender, smoking status, drinking status, BMI, tumor position, tumor differentiation, tumor stage, and chemotherapy, where appropriate.

## Discussion

In this study, we reported a significant association of the overall survival in a cohort of 408 surgically-treated CRC patients with a genetic variant, rs1954727, in *ANGPT1*, a pivotal gene in the VEGF-independent angiogenic signaling pathway. Furthermore, we identified a plausible interaction between this SNP and chemotherapy on modulating CRC patient survival.

Angiogenesis inhibitors targeting the VEGF signaling pathway, such as bevacizumab, sunitinib, and sorafenib, have demonstrated significant therapeutic efficacy in various cancers and been approved by the U.S. Food and Drug Administration in cancer treatment [24]. However, these VEGF-targeting anti-angiogenesis treatments frequently result in only transient responses in cancer patients, primarily owing to the two modes of resistance to angiogenesis inhibition, adaptive resistance and intrinsic non-responsiveness [13]. Both modes of resistance can be attributed to the inherent heterogeneity of genetically unstable tumor cells, the presence of redundant angiogenic factors, and the recruitment of hematopoietic cells and inflammatory cells into the tumor mass [13]. It is conceivable that therapeutic approaches simultaneously targeting multiple angiogenic factors, inflammatory pathways, and metastasis processes could induce more clinically meaningful responses [25]. The ANGPT-TIE pathway presents an attractive opportunity for such a therapeutic intervention as it is not only crucial for angiogenesis and vascular homeostasis in an VEGF-independent manner, but it also provides an important link between the angiogenesis and the inflammation pathways [25].

Human ANGPT-TIE protein family is composed of three ligands, ANGPT1, ANGPT2 and ANGPT4, as well as two receptors, TIE1 and TIE2. ANGPT1 is the most important mediator of VEGF-independent pro-angiogenic signaling pathway, which activates the downstream PI3K/AKT pathway through the interaction with the TIE2 protein [26], [27]. ANGPT2 inhibits ANGPT1 binding to the TIE2 receptor and prevents its activation [28]. Dynamic balance and sequential expression of ANGPTs and VEGF is required for maintaining angiogenesis [29], [30]. The up-regulation of ANGPT1 has been investigated in many cancers, suggesting that ANGPT1 is strongly correlated with tumor malignancy [25]. However, several studies have reported an inhibiting effect of ANGPT1 on the pathologic vascular expansion, indicating that ANGPT1 may also function as a tumor suppressor in several cancers, including CRC [31], [32], [33], [34]. These findings were consistent with the reports of several independent studies showing that higher ratio of ANGPT2/ANGTP1 was associated with poor prognosis of multiple malignancies including CRC [25]. These paradoxical findings suggest the effects of ANGPT1 on tumor characteristics and prognosis might be cancer-specific and also dependent on other angiogenesis-related genes.

Genetic variations in ANGPT genes may lead to altered gene production and result in activation/inactivation of the gene. SNPs in *ANGPT1* gene have been associated with the risk of diseases such as autoimmune diseases, juvenile idiopathic arthritis, and portopulmonary hypertension [35], [36]. However, to date, there has been no study reporting an association of *ANGPT1* SNPs with the risk and clinical outcome of cancers. In this study, we identified an *ANGPT1* SNP, rs1954727, which may predict the overall survival of CRC patients after surgery. This SNP is located in the 3′ UTR of *ANGPT1* gene. Functional SNPs in this region may affect messenger RNA stability or microRNA binding. A bioinformatics search of the PolymiRTS database did not identify any potential miRNA binding site in the immediate surrounding region of this SNP [37]. However, a further search led to the identification of four SNPs in the 3′UTR of *ANGPT1* gene that are located within putative miRNA binding regions (data not shown). The nearest SNP is about 600 nucleotides away from rs1954727. Functional characterizations such as real-time PCR followed by luciferase assay will be needed to determine whether rs1954727 has any direct physiological impact on messenger RNA expression of *ANGPT1*.

In stratified analysis, we found the protective effect conferred by rs1954727 was more prominent in patients receiving chemotherapy (Table S1). Previous studies have reported a significantly increased antitumor activity upon the joint use of ANGPT1 antibody and chemotherapy in treating solid tumors including CRC [38]. These lines of evidence led us to further evaluate the modulating effect of chemotherapy on patient survival stratified by the genotypes of rs1954727. Consistent with the finding that the variant genotypes of rs1954727 conferred better overall survival in chemotherapy-treated patients, we found the improved survival conferred by chemotherapy remained evident in patients with the heterozygous genotype of rs1954727 (Table 4). We also noted that in patients with the homozygous variant genotype of rs1954727, the *P* value was not significant (P = 0.297), although the improved overall survival conferred by chemotherapy was much more prominent (HR = 0.19). This may be due to an unstable estimate resulting from the small patient number in this group (only 1 death in patients without chemotherapy, Table 3).Future studies with larger sample size are needed to provide sufficient statistical power to the analysis of the interactions between rs1954727 and chemotherapy on patient survival.

There are several strengths in this study. The patients were enrolled from Xi'an and adjacent area, a region that is highly attractive for conducting population-based research due to the geographical stability with low mobility rate. Also, the patients analyzed in this study were highly homogenous, in that all patients had adenocarcinoma and were surgically treated to remove the primary tumors. Additionally, all chemotherapy treatments were started within 2 months of surgery and almost 80% of the patients received chemotherapy. The highly homogenous patient characteristics and treatments, as well as low rate of patient loss to follow-up, greatly reduced the confounding effects of the heterogeneous therapeutics modalities in many other similar biomarker studies of CRC prognosis. The limitations of our study include the generalizability issue, because our study was restricted to Han Chinese. Further evaluation is necessary to determine whether these findings can be generalized to other ethnic groups. Moreover, the moderate sample size limited the validity of some stratified analyses with small sample size. We analyzed the study power for the main effect analysis as well as the chemotherapy-stratified analysis. Under the condition of an additive genetic model (used in the analysis of this study) with monotonic effect, an MAF of 43.5% for rs1954727, the only significant SNP identified in this study, 23.0% of death rate among the total study population, a median time to death of 15.2 months in deceased patients and a median time of follow-up of 23.9 months in censored patients, we have approximately 82% power to detect an HR of 0.6 (heterozygous genotype vs. homozygous wild-type genotype) and 0.4 (homozygous variant genotype vs. wild-type genotype). The power is slightly higher if using a trend test. When the analysis is restricted to the patients receiving chemotherapy, our power is approximately 70% to detect the aforementioned effect sizes. However, our power is limited when the analysis is restricted to the 89 patients without chemotherapy or some other non-significant SNPs in this study that have a low MAF, and the results of these under-powered analyses are exploratory and should be interpreted with caution. Nonetheless, the enrollment of this population is still ongoing with a low rate of patient loss, which will enable us to obtain higher statistical power for in-depth analyses in the future.

In conclusion, our finding suggests that a genetic polymorphism in *ANGPT1* gene is associated with a significantly better overall survival in CRC patients after surgery. Future studies with larger populations are needed to validate this finding.

## Supporting Information

Table S1 **Association of rs1954727 in ANGPT1with overall survival in CRC patients stratified by host characteristics.**
(DOC)
