# Supplementary material for: Genetic Polymorphism in a VEGF-Independent Angiogenesis Gene ANGPT1 and Overall Survival of Colorectal Cancer Patients after Surgical Resection
Source: PLoS One. 2012 Apr 4;7(4):e34758. doi: 10.1371/journal.pone.0034758 (PMC3319640; doi:10.1371/journal.pone.0034758)
Supplement: Table S1 — Association of rs1954727 in ANGPT1with overall survival in CRC patients stratified by host characteristics. (DOC) [file pone.0034758.s001.doc]

| **Table S1. Association of rs1954727 in *ANGPT1*with overall survival in CRC patients stratified by host characteristics** | | | | | |
| --- | --- | --- | --- | --- | --- |
| **Variables** | **Strata** | **Genotype^a^** | **Death/total** | **HR (95% CI)^b^** | ***P* value** |
| Age | Younger ( ≤ 61) | WW | 16/ 62 | 1(reference) |  |
|  |  | WV | 32/100 | 1.35(0.68-2.66) | 0.387 |
|  |  | VV | 6/ 48 | 0.43(0.14-1.31) | 0.139 |
|  |  | *P* for trend |  |  | 0.300 |
|  | Older( > 61) | WW | 17/ 71 | 1(reference) |  |
|  |  | WV | 20/ 92 | 0.55(0.26-1.18) | 0.127 |
|  |  | VV | 3/ 32 | **0.12(0.03-0.60)** | **0.010** |
|  |  | *P* for trend |  |  | **0.006** |
|  |  |  |  |  |  |
| Gender | Male | WW | 17/ 58 | 1(reference) |  |
|  |  | WV | 21/ 79 | 0.86(0.38-1.95) | 0.726 |
|  |  | VV | 4/ 40 | **0.20(0.05-0.75)** | **0.017** |
|  |  | *P* for trend |  |  | **0.012** |
| . | Female | WW | 16/ 75 | 1(reference) |  |
|  |  | WV | 31/113 | 0.97(0.50-1.86) | 0.918 |
|  |  | VV | 5/ 40 | 0.60(0.17-2.11) | 0.430 |
|  |  | *P* for trend |  |  | 0.449 |
|  |  |  |  |  |  |
| Educational level | Up to high school | WW | 14/ 53 | 1(reference) |  |
|  |  | WV | 25/ 88 | 1.29(0.60-2.77) | 0.509 |
|  |  | VV | 4/ 37 | **0.28(0.08-0.94)** | **0.040** |
|  |  | *P* for trend |  |  | 0.098 |
| . | College degree or higher | WW | 17/ 62 | 1(reference) |  |
|  |  | WV | 22/ 76 | 0.82(0.41-1.64) | 0.573 |
|  |  | VV | 4/ 32 | 0.35(0.11-1.16) | 0.086 |
|  |  | *P* for trend |  |  | 0.059 |
|  |  |  |  |  |  |
| Body mass index (BMI) | BMI ≤ 22.7 | WW | 14/ 75 | 1(reference) |  |
|  |  | WV | 24/ 92 | 0.91(0.41-2.03) | 0.822 |
|  |  | VV | 4/ 38 | **0.20(0.04-0.97)** | **0.045** |
|  |  | *P* for trend |  |  | 0.062 |
|  | BMI > 22.7 | WW | 19/ 58 | 1(reference) |  |
|  |  | WV | 28/100 | 0.67(0.35-1.29) | 0.228 |
|  |  | VV | 5/ 42 | **0.32(0.11-0.92)** | **0.035** |
|  |  | *P* for trend |  |  | **0.025** |
|  |  |  |  |  |  |
| Smoking status | Never smoker | WW | 25/100 | 1(reference) |  |
|  |  | WV | 39/131 | 1.00(0.56-1.79) | 0.997 |
|  |  | VV | 6/ 57 | **0.28(0.11-0.73)** | **0.009** |
|  |  | *P* for trend |  |  | **0.021** |
| . | Ever smoker | WW | 8/ 33 | 1(reference) |  |
|  |  | WV | 13/ 61 | 0.48(0.16-1.41) | 0.180 |
|  |  | VV | 3/ 23 | 0.15(0.00-10.1) | 0.379 |
|  |  | *P* for trend |  |  | 0.298 |
|  |  |  |  |  |  |
| Drinking status | Never drinker | WW | 28/120 | 1(reference) |  |
|  |  | WV | 44/170 | 0.92(0.54-1.54) | 0.740 |
|  |  | VV | 8/ 72 | **0.35(0.15-0.84)** | **0.018** |
|  |  | *P* for trend |  |  | **0.025** |
|  | Ever drinker | WW | 5/ 13 | 1(reference) |  |
|  |  | WV | 8/ 22 | 0.93(0.12-7.14) | 0.944 |
|  |  | VV | 1/ 8 | NA |  |
|  |  | *P* for trend |  |  |  |
|  |  |  |  |  |  |
| Chemotherapy | No | WW | 7/31 | 1(reference) |  |
|  |  | WV | 14/ 43 | 0.40(0.11-1.42) | 0.155 |
|  |  | VV | 1/ 15 | NA |  |
|  |  | *P* for trend |  |  |  |
|  | Yes | WW | 26/102 | 1(reference) |  |
|  |  | WV | 38/149 | 0.89(0.51-1.56) | 0.689 |
|  |  | VV | 8/ 65 | **0.33(0.14-0.79)** | **0.012** |
|  |  | *P* for trend |  |  | **0.012** |
|  |  |  |  |  |  |
| Tumor position | Colon | WW | 17/ 69 | 1(reference) |  |
|  |  | WV | 23/ 83 | 0.76(0.36-1.58) | 0.458 |
|  |  | VV | 4/ 38 | **0.09(0.02-0.40)** | **0.002** |
|  |  | *P* for trend |  |  | **0.008** |
|  | Rectum | WW | 16/ 64 | 1(reference) |  |
|  |  | WV | 29/109 | 0.73(0.37-1.45) | 0.373 |
|  |  | VV | 5/ 42 | 0.48(0.14-1.71) | 0.261 |
|  |  | *P* for trend |  |  | 0.116 |
|  |  |  |  |  |  |
| Tumor differentiation | Poor and moderate | WW | 26/ 97 | 1(reference) |  |
|  |  | WV | 44/151 | 0.90(0.53-1.53) | 0.710 |
|  |  | VV | 7/ 57 | **0.32(0.13-0.78)** | **0.012** |
|  |  | *P* for trend |  |  | **0.016** |
|  | Well | WW | 7/ 36 | 1(reference) |  |
|  |  | WV | 8/ 41 | 0.56(0.14-2.27) | 0.413 |
|  |  | VV | 2/ 23 | NA |  |
|  |  | *P* for trend |  |  |  |
|  |  |  |  |  |  |
| Tumor stage | Stage 0-2 | WW | 15/ 90 | 1(reference) |  |
|  |  | WV | 20/114 | 0.74(0.34-1.61) | 0.446 |
|  |  | VV | 4/ 52 | 0.37(0.11-1.26) | 0.111 |
|  |  | *P* for trend |  |  | 0.065 |
|  | Stage 3-4 | WW | 18/ 43 | 1(reference) |  |
|  |  | WV | 32/ 78 | 0.63(0.32-1.26) | 0.189 |
|  |  | VV | 5/ 28 | **0.16(0.04-0.65)** | **0.011** |
|  |  | *P* for trend |  |  | **0.003** |
| Note: The significant *P* values (≤0.05) were in bold. | | | | | |
| a WW, homozygous wild-type genotype; WV heterozygous genotype; VV, homozygous variant genotype. | | | | | |
| b Adjusted for age, gender, smoking status, drinking status, BMI, tumor position, tumor differentiation, tumor stage, and chemotherapy, where appropriate. | | | | | |
